# Supplementary material for: Linking genetic and phenotypic bedaquiline resistance in Mycobacterium tuberculosis strains from Georgia
Source: PLoS One. 2025 Jul 15;20(7):e0326794. doi: 10.1371/journal.pone.0326794 (PMC12262877; doi:10.1371/journal.pone.0326794)
Supplement: S1. Table — (DOCX) [file pone.0326794.s001.docx]

S1 Table. List of mutations in *atpE, pepQ* and Rv0678, isolate quantity, corresponding MICs and lineage distribution of study isolates.

|  | ≤0.03 ɥg/mL | 0.06 ɥg/mL | 0.12 ɥg/mL (borderline resistance) | 0.25 ɥg/mL (resistance cut-off) | 0.5 ɥg/mL | L2 | L3 | L4 |
| --- | --- | --- | --- | --- | --- | --- | --- | --- |
| Ala196Val^1^ | 3 | - | - | - | - | 3 | - | - |
| Ser52Phe ^3^ * | 1 | - | - | - | - | 1 | - | - |
| Asp151Gly ^1^ * | 1 | - | - | - | - | 1 | - | - |
| Glu129Gln ^1^ | 1 | - | - | - | - | 1 | - | - |
| Gly285Asp ^1^ | 1 | - | - | - | - | 1 | - | - |
| His100Tyr ^1^ | 1 | - | - | - | - | 1 | - | - |
| Val336Ala ^1^ | 1 | - | - | - | - | 1 | - | - |
| Met73Val ^3^ * | 1 | - | - | - | - | 1 | - | - |
| Arg86Leu ^1^ | 1 | - | - | - | - | - | 1 | - |
| Ala263Val^1^ ; Asp26Gly^1^ * | 1 | - | - | - | - | - | - | 1 |
| Phe76Leu ^2^ * | 7 | - | - | - | - | - | - | 7 |
| Gly11_Pro14del ^3^; Asp15Tyr ^3^ * | 1 | - | - | - | - | - | - | 1 |
| Gly309Glu ^1^ | 1 | - | - | - | - | - | - | 1 |
| Ile202Met ^1^ | 1 | - | - | - | - | - | - | 1 |
| Ile28Met ^1^ | 1 | - | - | - | - | - | - | 1 |
| Val134Leu ^1^ | 2 | - | - | - | - | - | - | 2 |
| Ala71Val ^3^ * | 1 | - | - | - | - | - | - | 1 |
| Gln262Arg ^1^ * | 1 | 4 | - | - | - | - | - | 5 |
| Gln9* ^3^ * | - | 1 | - | - | - | - | - | 1 |
| Ser99Arg ^1^ | - | 2 | - | - | - | - | - | 2 |
| Thr315Arg ^1^ * | - | 1 | - | - | - | - | - | 1 |
| Ala55Thr ^1^ * | - | 1 | - | - | - | 1 | - | - |
| Met1fs ^3^ * | - | 1 | - | - | - | 1 | - | - |
| Arg89Leu ^3^ * | - | 1 | - | - | - | 1 | - | - |
| Arg96Trp ^3^; Leu122Met; ^3^ * | - | 2 | - | - | - | 2 | - | - |
| Leu44Pro ^3^ * | - | 1 | - | - | - | 1 | - | - |
| Met139Thr ^3^ * | - | 1 | - | - | - | 1 | - | - |
| Phe93Leu ^3^ * | - | 1 | 5 | - | - | 6 | - | - |
| Ala86Ser ^3^ * | - | - | 1 | - | - | 1 | - | - |
| Asn70Ile ^3^ | - | - | 1 | - | - | 1 | - | - |
| Asp47fs ^3^ | - | - | 1 | - | - | 1 | - | - |
| Ala102Asp ^3^; Leu95Ser ^3^ * | - | - | - | 1 | - | 1 | - | - |
| Tyr92Cys ^3^ * | - | - | - | 1 | 1 | - | - | 2 |
| ^1^ *pepQ*; ^2^ *atpE*; ^3^ *Rv0678;* * Isolates had and an additional mutations in same and/or *pepQ*, *atpE*, *Rv0678* genes below 20% of allele frequency. | | | | | | | | |
