# Supplementary material for: Linking genetic and phenotypic bedaquiline resistance in Mycobacterium tuberculosis strains from Georgia
Source: PLoS One. 2025 Jul 15;20(7):e0326794. doi: 10.1371/journal.pone.0326794 (PMC12262877; doi:10.1371/journal.pone.0326794)
Supplement: S2 Table — (DOCX) [file pone.0326794.s002.docx]

S2 Table. Study isolates (69) with corresponding accession number of the European Nucleotide Archive (ENA), mutations with variant frequency, inclusion in WHO catalogue (second edition) and MIC values (resistance cut-off: 0.12mg/L –borderline; 0.25mg/L resistance)

| **Accession number** | ***atpE* (freq.%)** | ***pepQ* (freq.%)** | **Rv0678 (freq.%)** | **WHO catalogue** | **Drug** | **MIC** |
| --- | --- | --- | --- | --- | --- | --- |
| SAMEA7157494 | - | - | Met1fs(99%) | Not in the catalgue |  | 0.06 |
| SAMEA115879598 | - | His100Tyr(44.1%) | - | Not in the catalgue |  | 0.03 |
| SAMEA7157182 | - | - | Phe93Leu(99.3%) | Uncertain significance | BDQ; CFZ | 0.12 |
| SAMEA115879599 | Phe76Leu(99.4%) | - | - | Not in the catalogue |  | 0.03 |
| SAMEA115879600 | - | - | Phe93Leu(98.4%) | Uncertain significance | BDQ; CFZ | 0.12 |
| SAMEA7156796 | - | Arg50His(3.1%) | Ser52Phe(96.9%)  Val1? (start_lost)(4.6%) | Uncertain significance | BDQ; CFZ | 0.03 |
| SAMEA7157187 | - | - | Asp47fs(99%) | Associated w R | BDQ | 0.12 |
| SAMEA7157183 | - | - | Phe93Leu(99.1%) | Uncertain significance | BDQ; CFZ | 0.12 |
| SAMEA7157195 | - | - | Phe93Leu(99.2%) | Uncertain significance | BDQ; CFZ | 0.12 |
| SAMEA7157116 | - | - | Tyr92Cys(99.2%) | Not in the catalgue |  | 0.25 |
| SAMEA115879601 | - | - | Asn70Ile(95.6%) | Uncertain significance | BDQ | 0.12 |
| SAMEA7157507 | - | - | Ala86Ser(98.9%) | Not in the catalogue |  | 0.12 |
| SAMEA115879602 | - | - | Gly11_Pro14del (33.4%) Asp15Tyr(33.3%) | Not in the catalogue |  | 0.03 |
| SAMEA115879603 | - | Arg164Trp(4.4%) Arg160Cys(4.9%) | Phe93Leu(98.7%) | Uncertain significance | BDQ; CFZ | 0.06 |
| SAMEA7157382 | - | Val336Ala(99.3%) | - | Not in the catalogue |  | 0.03 |
| SAMEA12854060 | Phe76Leu(99.4%) | - | - | Not in the catalogue |  | 0.03 |
| SAMEA12851245 | - | Ile202Met(98.2%) | - | Not in the catalogue |  | 0.03 |
| SAMEA7157235 | - | - | Met73Val(98.1%) | Not in the catalogue |  | 0.03 |
| SAMEA7157575 | - | Glu129Gln(98.2%) | Val150Leu(2.7%) | Not in the catalogue |  | 0.03 |
| SAMEA7157588 | - | - | Leu44Pro(80.1%) **Glu49fs(7.8%)** | Associated w R | CFZ | 0.06 |
| SAMEA12854845 | - | Gln262Arg(98.4%) | Glu163Asp(5%) | Not in the catalogue |  | 0.06 |
| SAMEA12854832 | - | Ala196Val(98.2%) | - | Not in the catalogue |  | 0.03 |
| SAMEA12852275 | Val39Ala(5.2%) | Ala263Val(97.6%) Asp26Gly(5.7%) | Val150Leu(6.1%) | Not in the catalogue |  | 0.03 |
| SAMEA12854904 | Phe76Leu(98.6%) | - | - | Not in the catalogue |  | 0.03 |
| SAMEA12855315 | - | Val134Leu(98%) | - | Not in the catalogue |  | 0.03 |
| SAMEA12854786 | Phe76Leu(98.3%) | - | - | Not in the catalogue |  | 0.03 |
| SAMEA7157196 | - | - | Phe93Leu(98.9%) | Uncertain significance | BDQ; CFZ | 0.12 |
| SAMEA12854519 | - | Gln262Arg(97%) | - | Not in the catalogue |  | 0.06 |
| SAMEA12855289 | - | Arg86Leu(97.3%) | - | Not in the catalogue |  | 0.03 |
| SAMEA12854867 | - | Ile28Met(98.7%) | - | Not in the catalogue |  | 0.03 |
| SAMEA7157117 | - | - | Tyr92Cys(98.9%) | Not in the catalogue |  | 0.5 |
| SAMEA12854852 | Phe76Leu(98.4%) | - | - | Not in the catalogue |  | 0.03 |
| SAMEA12854149 | - | Gly309Glu(97.3%) | - | Not in the catalogue |  | 0.03 |
| SAMEA12855448 | - | - | Ala71Val(98.5%) Glu163Asp(3.9%) | Not in the catalogue |  | 0.03 |
| SAMEA12851782 | Phe76Leu(96.8%) | - | - | Not in the catalogue |  | 0.03 |
| SAMEA12854332 | - | Gln262Arg(98.5%) | - | Not in the catalogue |  | 0.06 |
| SAMEA12854777 | - | Gln262Arg(98.3%) | Glu163Asp(4.2%) | Not in the catalogue |  | 0.06 |
| SAMEA12855094 | - | Ala55Thr(98.4%) | - | Not in the catalogue |  | 0.06 |
| SAMEA12854352 | - | Ala196Val(98.8%) | - | Not in the catalogue |  | 0.03 |
| SAMEA12854428 | - | Thr315Arg(98.1%) | Val150Leu(4.9%) | Not in the catalogue |  | 0.06 |
| SAMEA12854512 | - | Ser99Arg(97.9%) | - | Not in the catalogue |  | 0.06 |
| SAMEA12851961 | - | Gln262Arg(98.2%) | - | Not in the catalogue |  | 0.03 |
| SAMEA7157033 | - | Glu360* (3.9%) | Leu95Ser(78.1%) Ala102Asp(15.1%)  Glu28*(9.8%) | Uncertain significance | BDQ; CFZ | 0.25 |
| SAMEA12855114 | - | Ser99Arg (98.4%) | - | Not in the catalogue |  | 0.06 |
| SAMEA12854256 | - | Ala196Val(98.7%) | - | Not in the catalogue |  | 0.03 |
| SAMEA12852776 | - | Val134Leu(98.2%) | - | Not in the catalogue |  | 0.03 |
| SAMEA115879604 | - | - | Arg96Trp(98.7%) Leu122Met(98.7%) | Not in the catalogue |  | 0.06 |
| SAMEA12854379 | Phe76Leu(98.8%) | - | - | Not in the catalogue |  | 0.03 |
| SAMEA115879605 | - | Gly285Asp(975%) | - | Not in the catalogue |  | 0.03 |
| SAMEA115879606 | Val39Ala(5.3%) | Asn40Ile(3.4%) | Gln9*(98.8%) | Not in the catalogue |  | 0.06 |
| SAMEA115879607 | - | Asp151Gly(99.2%) | Val150Leu(3.1%) | Not in the catalogue |  | 0.03 |
| SAMEA115879608 | - | - | Met139Thr(52.5%) | Not in the catalogue |  | 0.06 |
| SAMEA115879609 | - | - | Arg89Leu(99.1%) | Not in the catalogue |  | 0.06 |
| SAMEA115879610 | - | - | Arg96Trp(76.5%) Leu122Met(80.4%) Leu114Arg(18.5%) | Not in the catalogue |  | 0.06 |
| SAMEA115432917 | - | - | **Ile67fs(49.4%)**  Arg38Gly(15.3%) Arg135Trp(11.7%)  Trp42Arg(7.8%) Ala59Glu(7.9%) Met139Thr(4.7%) | Associated w R | BDQ | 0.25 |
| SAMEA115432916 | - | - | Leu32Ser(98.9%) | Associated w R | BDQ;CFZ | 0.5 |
| SAMEA115879611 | - | - | Ala102Asp(99.1%) | Not in the catalogue |  | 0.5 |
| SAMEA115879612 | - | - | - | NA |  | 0.12 |
| SAMEA115879613 | - | - | - | NA |  | 0.12 |
| SAMEA115879614 | - | - | Glu163Asp(3.8%) | Not in the catalogue |  | 0.12 |
| SAMEA115879615 | - | - | - | NA |  | 0.25 |
| SAMEA115879616 | - | - | - | NA |  | 0.25 |
| SAMEA115879617 | - | - | - | NA |  | 0.25 |
| SAMEA115432889 | Ile66Met(99.1%) | - | - | Associated w R | BDQ | 0.5 |
| SAMEA115879618 | **Ala63Pro(24.2%)** | - | Ile108fs(25%) | Associated w R | BDQ | 0.5 |
| SAMEA115879619 | - | - | - | NA |  | 0.06 |
| SAMEA115432888 | Ile66Met(99%) | - | - | Associated w R | BDQ | 0.5 |
| SAMEA115879620 | - | - | - | NA |  | 0.03 |
| SAMEA115879621 | - | - | - | NA |  | 0.03 |
